# Analysis of the Efficacy and Safety of Avatrombopag Combined With MSCs for the Treatment of Thrombocytopenia After Allogeneic Hematopoietic Stem Cell Transplantation

**DOI:** 10.3389/fimmu.2022.910893

**Published:** 2022-05-27

**Authors:** Lidan Zhu, Jia Liu, Peiyan Kong, Shichun Gao, Lu Wang, Huanfeng Liu, Cheng Zhang, Li Gao, Yimei Feng, Ting Chen, Lei Gao, Xi Zhang

**Affiliations:** Medical Center of Hematology, The Second Affiliated Hospital, Army Medical University, Chongqing, China

**Keywords:** avatrombopag, allogeneic hematopoietic stem cell transplantation (AlloHCT), safety, thrombocytopenia, umbilical cord MSCs

## Abstract

Platelet graft failure (PGF) is a frequent and serious complication after Allogeneic hematopoietic stem cell transplantation (allo-HSCT) and lacks effective treatment strategies, which could affect the prognosis of patients and even cause death. The exact underlying mechanism of PGF remains unclear, and lacks standard treatment. Here, we conduct a retrospective study to evaluate the efficacy and safety of avatrombopag combined with mesenchymal stem cells (MSCs) in 16 patients with thrombocytopenia after allo-HSCT. Patients were administered the following treatment regimen: 20 mg/d avatrombopag; if the PLT count was less than 50×10^^^9/L for at least 2 weeks, the dose was increased to 40 mg/d; if the PLT count was 200-400×10^^^9/L, the dose was reduced; and if the PLT count was greater than 400×10^9/L, avatrombopag was terminated. Umbilical cord MSCs (1×10^^^6 cells/kg) infusion was performed every week for 4-6 weeks. Among the 16 patients, 13 patients (81.3%) achieved a complete response (CR), 2 patients (12.5%) got a partial response (PR), and 1 patient (6.3%) had no response (NR). The median time to obtain CR was 32 (7-426) days after treatment with avatrombopag combined with umbilical cord MSCs. The time to reach 20×10^^^9/L≤ PLT <50×10^^^9/L in the 2 patients with PR was 52 and 230 days after treatment, respectively. One patient had a severe pulmonary infection and died of cytomegalovirus pneumonia. Overall, our results indicated that combination of avatrombopag with MSCs can promote platelet recovery after transplantation, thereby improving the survival rate of patients and improving the quality of life of patients after transplantation, and providing a new method and strategy for the treatment of thrombocytopenia after allo-HSCT.

## Introduction

Allogeneic hematopoietic stem cell transplantation (allo-HSCT) is an effective strategy for the treatment of benign and malignant hematological diseases. Which could be used to reconstruct the patients hematopoietic and immune functions, and the stable implantation of donor HSCs is the basis for the success of allo-HSCT (1). The successful engraftment of HSCs defined as the recovery of neutrophils, erythroid cells and platelets. Neutrophil engraftment is defined as a neutrophil count exceeding 0.5×10^^^9/L for 3 consecutive days, platelet engraftment is defined as a platelet count no lower than 20×10^^^9/L for 7 consecutive days without platelet transfusion, and red blood cell engraftment is defined as a hemoglobin concentration no less than 70 g/L without blood transfusion (2). The recovery of neutrophils after transplantation is relatively fast, with a median time of 10-17 days and often within 28 days. Platelet recovery time varies significantly, ranging from 2 weeks to several months or even 1 year after transplantation (3).

Platelet graft failure is a frequent and serious complication after allo-HSCT, and prolonged thrombocytopenia after transplantation is major type which could affect the long-term survival of patients. Thrombocytopenia after HSCT could be divided into 2 categories: prolonged or prolonged isolated thrombocytopenia (PT/PIT) – all blood cells in the peripheral blood except platelets (PLT) recover to normal levels after HSCT, and PLT counts are lower than 20×10^^^9/L for longer than 3 months, without relapse and clear cause (4); and secondary failure of platelet recovery (SFPR) – PLT≥50×10^^^9/L for 7 consecutive days after HSCT without blood transfusion support, then PLT ≤ 20×10^^^9/L occurred for more than 7 consecutive days after primary PLT recovery or PLT transfusion required (5). The risk factors for thrombocytopenia after HSCT include graft versus host disease (GVHD), number of HSCs, anti-HLA antibodies (donor-specific antibodies) (6), previous alloimmunization and strength of the pretreatment regimen, cytomegalovirus (CMV), graft source, low PLT before pretreatment, iron overload, etc. (7). For posttransplant thrombocytopenia, there is currently a lack of standard treatment regimens, and many regimens are offered through clinical studies and generally have a long intervention period. Thrombopoietin, intravenous gamma globulin, epigenetic regulation drugs, and anti-CD20 monoclonal antibodies are often used for treatment, but with limited efficacy. A number of studies have found that immune abnormalities in the bone marrow microenvironment, endothelial cell damage, mesenchymal stems (MSCs) damage, and an oxygen metabolism imbalance can lead to HSC damage, subsequently leading to the occurrence of primary graft failure (PGF). MSCs promote the proliferation and differentiation of HSCs by secreting a variety of cytokines, thus enhancing hematopoietic function, providing hematopoietic support, and directly participating in or indirectly promoting vascular regeneration in injured tissues and organs (8). Avatrombopag is a second-generation thrombopoietin (TPO) receptor agonist, which was used to treat chronic immune thrombocytopenia with earlier and long-lasting response (9). However, the efficacy and safety of avatrombopag for the treatment of PT after allo-HSCT are still unclear. As an important component of the bone marrow hematopoietic microenvironment, MSCs secrete a variety of cytokines that support hematopoiesis, promote hematopoietic reconstruction after transplantation, and accelerate the recovery of hematopoietic function in the body; but the clinical efficacy is not stable restricted by different source and different passages. Our previous study demonstrated that Umbilical cord blood MSC was an optimal source which could decrease the occurrence of cGVHD and accompanied by leading to the acquisition of immune tolerance. Which suggested that Umbilical cord blood MSC showed superior capacity in treating the complication after all-HSCT, whether the combination of avatrombopag and MSCs promote PLT engraftment after transplantation through a synergistic effect was worth to figure out.

In this study, we summarize the treatment of 16 patients with PT after allo-HSCT using avatrombopag combined with umbilical cord MSCs in our center to explore the efficacy and safety, and our results will provide a new method and strategy for the treatment of thrombocytopenia after allo-HSCT.

## Cases and Methods

### Case Data

From August 11, 2020, to December 21, 2021, 16 patients with thrombocytopenia after allo-HSCT were treated with avatrombopag combined with MSCs in our department. All patients had complete donor engraftment; 13 patients had SFPR, and 3 patients had PT. The above patients had no complications, such as severe infection and GVHD, and the disease was in complete remission. This study was approved by the Ethics Committee of the Second Affiliated Hospital of Army Medical University.

### Treatment Plan

#### Transplantation Regimen

Patients undergoing haploidentical related donor HSCT (haplo-HSCT) received pretreatment with the MeCCNU+BU+CTX+Ara-C+ATG regimen (semustine, 0.2 g/m^2^ × 1 d; busulfan, 0.8 mg/kg q6 h × 3 d; cyclophosphamide, 1.8 g/m^2^ × 2 d; cytarabine, 2 g/m^2^ q12 h × 2 d; and anti-human thymus immunoglobulin, 2.5 mg/kg × 4 d); sibling matched donor (SMD)-HSCT patients were treated with the BU+CY regimen (busulfan 0.8 mg/kg q 6 h×4 d and cyclophosphamide 60 mg/kg×2 d), and unrelated matched donor (UMD)-HSCT patients were treated with the BU+CY+ATG regimen (busulfan, 0.8 mg/kg q6 h×4 d; cyclophosphamide, 60 mg/kg×2 d; and anti-human thymus immunoglobulin, 2.5 mg/kg × 4 d).

Among the 16 patients who received a stem cell transfusion, 6 patients received peripheral blood HSCT. The average number of peripheral blood mononuclear cells (MNCs) transfused was 9.98 (6.4-12.8)×10^^^8/kg, and the average number of CD34^+^ cells was 4.87 (2.6~7)×10^^^6/kg. Ten patients received peripheral blood combined with bone marrow transplantation. The average number of peripheral blood MNCs transfused was 10.94 (8.4~15.4) ×10^^^8/kg, and the average number of CD34^+^ cells was 6.14 (3.68~8.7) ×10^^^6/kg. The average number of bone marrow nucleated cells was 3.25 (1.88~4.8) ×10^^^8/kg, and the average number of CD34^+^ cells was 0.79 (0.11~1.3) ×10^^^6/kg. All patients received a subcutaneous injection of 5~10 μg/kg/d recombinant human granulocyte colony stimulating factor 48-72 h after reinfusion of stem cells until hematopoietic reconstitution. Subcutaneous injections of recombinant human TPO were started on day 7 after transplantation at a dose of 300 U/kg/d. The drug was continuously administered for 14 days, or the drug was discontinued when the PLT count increased to 50×10^^^9/L.

#### GVHD Prevention and Treatment

The short-course tacrolimus (FK506) + methotrexate (MTX) + mycophenolate mofetil (MMF) regimen was administered to haplo-HSCT patients (10). Pretreatment with FK506 was started 7 days before HSCT (continuous intravenous infusion of 0.03 mg/kg/d FK506 for 24 h). After hematopoietic reconstitution, cyclosporine (CsA, 2.5mg/kg/d) was administered as a continuous intravenous infusion for 24 h. Oral cyclosporine (5 mg/kg q12 h) was administered after recovery of intestinal function. MTX was administered as follows: +1 day, 15mg/m^2^; and +3, +6, and +11 days, 10 mg/m^2^. MMF was administered 7 days before HSCT, with a total dosage of 600 mg/m^2^/day, twice per day; the daily dosage was reduced at +90 days and gradually reduced and stopped. SMD-HSCT patients received the CsA + MTX + MMF regimen to prevent GVHD. CsA (2.5 mg/kg/d) treatment was started from 1 day before HSCT *via* continuous intravenous infusion for 24 h. After hematopoietic reconstitution, CsA was administered orally at double the dose used for intravenous administration. MTX was administered on +1, +3, and +6 days, and UMD-HSCT patients required an additional dose of MTX on day +11. For acute GVHD, 2 mg/kg/d methylprednisolone was given as pulse therapy. If methylprednisolone treatment was ineffective, CD25 monoclonal antibody, ruxolitinib, or sirolimus were administered.

The avatrombopag and umbilical cord MSCs regimen was as follows. The initial dose of avatrombopag (20mg/d) was administered orally for at least 2 weeks; if PLT<50×10^^^9/L, the dose was increased to 40 mg/d; if PLT was 200-400×10^^^9/L, the dose was reduced by one dose level; and if PLT>400×10^^^9/L, avatrombopag was terminated. Umbilical cord MSCs were infused weekly (1×10^6^/kg) for 4-6 weeks.

### Efficacy Criteria

Complete response (CR) was defined as PLT≥50×10^^^9/L without PLT transfusion for 7 continuous days; partial response (PR) was defined as PLT of (20-50)×10^^^9/L without PLT transfusion for 7 continuous days; and no response (NR) was defined as the application of the maximum tolerated dose for 8 weeks and PLT<20×10^^^9/L or the need for PLT transfusion (7).

Adverse reaction assessment and follow-up adverse reactions were evaluated according to the National Cancer Institute Common Terminology Criteria for Adverse Events (NCI-CTCAE) version 5.0. The follow-up cut-off date was December 21, 2021, with weekly rechecking of complete blood count, liver and kidney function, and FK506/CsA concentrations within 3 months after transplantation and monthly rechecking of bone marrow aspiration biopsy, engraftment indicators (sex chromosomes, donor-recipient chimerism [short tandem repeat (STR), and immune function tests] for 6 months after transplantation. The above indicators were reviewed every 2 months from 6 months to 1 year after transplantation. One year after transplantation, the above indicators were evaluated every 3 months.

### Statistical Analysis

SPSS 19.0 software was used for the statistical analysis. Kaplan–Meier survival analysis was used to analyze the patient survival rate.

## Results

### General Information of the Patients

Among the 16 patients, 7 were females, and 9 were males, with a median age of 45.5 (16-58) years. There were 9 cases of acute myeloid leukemia, 3 cases of acute lymphoblastic leukemia, 3 cases of aplastic anemia, and 1 case of myelodysplastic syndromes. Among the patients, 3 underwent human lymphocyte antigen (HLA)-matched unrelated donor HSCT, 1 underwent 9/10 HLA-matched sibling donor HSCT, 2 underwent HLA-matched sibling donor HSCT, and 10 underwent haploidentical related donor HSCT (**Table 1**).

**Table 1:** Patients clinical characteristics (n = 16).

	Gender	Age	Disease types	disease status	Donor type	peripheral blood(MNC×10^8/kg/CD34+×10^6/kg)	Bone marrow(MNC×10^8/kg/CD34+×10^6/kg)	Neutrophil implantation(d)	Platelet implantation(d)	implantation	Transplantation related complications
1	Male	58	ALL	CR	UMD	6.4/3.9	–	21	25	100%	CMV
2	female	48	AA	–	haplo-HSCT	9.46/6.95	2.6/0.45	18	–	99.50%	Pulmonary infection
3	Male	39	AML	CR	UMD	9.36/2.6	–	22	16	100%	CMV
4	Male	45	AA	–	SMD	10.9/6.8	–	13	13	99.50%	–
5	Male	23	AML	CR	haplo-HSCT	15.4/6.1	2.1/0.97	22	20	100%	–
6	female	23	AML	CR	UMD	12.8/3.52	–	67	–	98%	Tuberculosis、GVHD
7	Male	48	AML	CR	haplo-HSCT	13.3/6.07	4.52/0.85	21	21	100%	Fungal infection
8	female	53	AML	CR	SMD9/10	10/7	–	13	13	99.50%	CMV
9	female	58	AML	CR	haplo-HSCT	11/6.1	2.4/1.07	25	–	99%	CMV、GVHD
10	Male	46	ALL	CR	haplo-HSCT	8.7/3.68	3.84/0.8	21	21	99.50%	CMV
11	female	54	AML	CR (MRD+)	SMD	10.4/5.4	–	20	20	100%	CMV
12	Male	34	AML	CR (MRD+)	haplo-HSCT	8.4/6.7	2.9/0.4	18	18	99.50%	CMV、Fungal infection
13	Male	34	MDS	–	haplo-HSCT	13.5/5.8	1.88/0.11	14	19	100%	encephalitis infection
14	female	30	AA	–	haplo-HSCT	10.1/8.7	4.8/1.3	21	37	100%	CMV、GVHD、hemorrhage
15	female	47	AML	CR	haplo-HSCT	10.9/4.2	3.3/1.1	15	15	100%	CMV、Fungal infection
16	Male	16	ALL	CR	haplo-HSCT	8.67/7.07	4.16/0.83	20	20	100%	CMV、GVHD

AML, acute myeloid leukemia; ALL, acute lymphoblastic leukemia; AA, aplastic anemia; MDS, myelodysplastic syndrome; UMD, Matched unrelated donor; SMD, matched sibling donor; haplo-HSCT, Haploidentical Hematopoietic Stem Cell Transplantation; CMV, cytomegalovirus; GVHD, graft versus host disease.

### Hematopoietic Reconstruction

**Figure 1:**
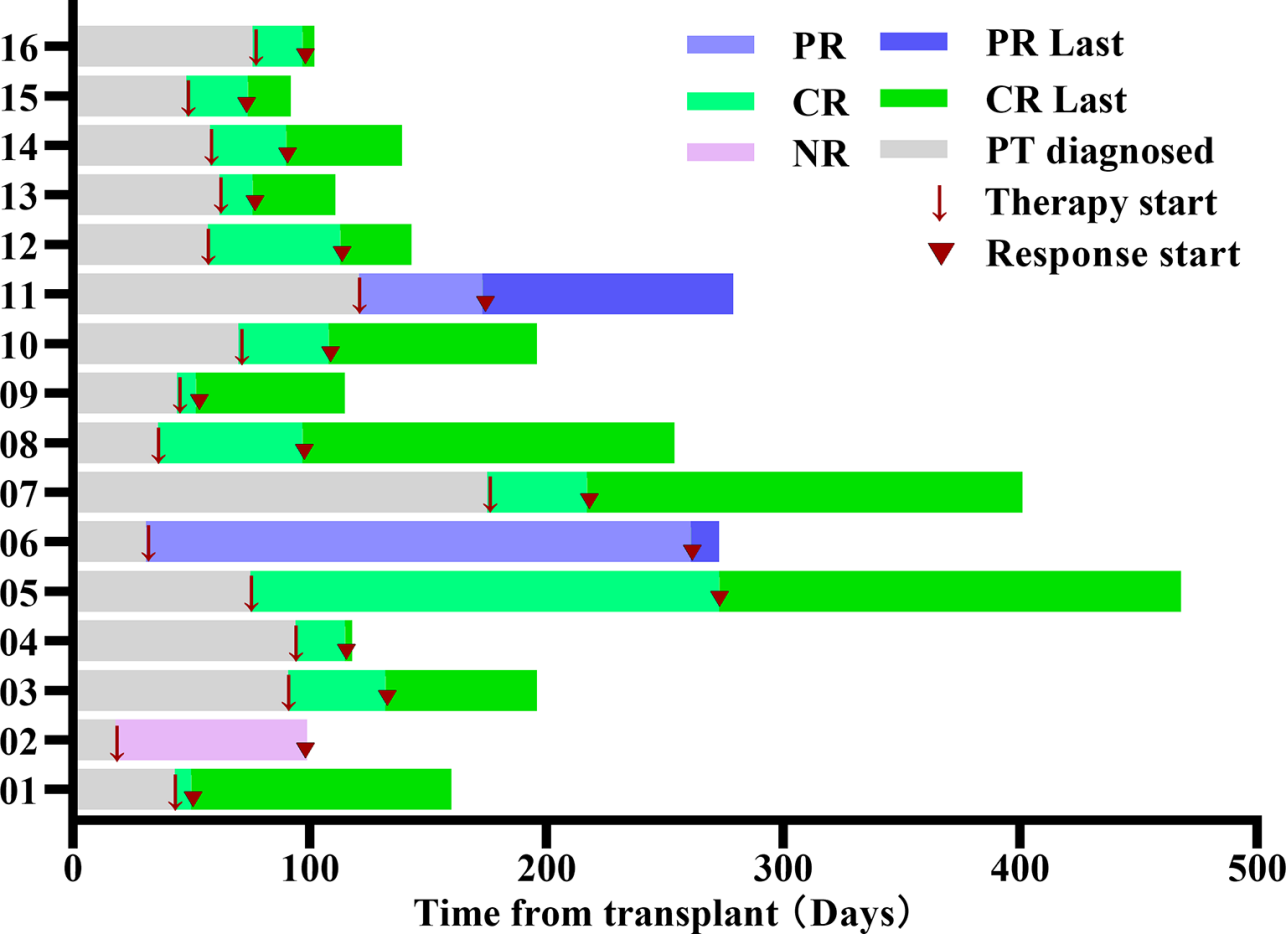
Swimmer plot for the response evaluable patients. Each bar represents an individual patient as designated. Arrow indicates the time of treatment. Triangle indicates the start time of clinical response. PR, partial response; CR, Complete response; NR, no response.

The median time of neutrophil recovery in the 16 patients was 20.5 (13-67) days, with 100% complete donor engraftment. Three patients diagnozed PT, and the median time was 28 (18-29) days after transplantation. Thirteen patients diagnozed SFPR; the time for platelet reconstitution was 20 (13-37) days, and the median time to reoccurrence of thrombocytopenia was 63 (35-133) days after transplantation. After the occurrence of thrombocytopenia, the median time to the initiation of treatment with avatrombopag was 66 (19-149) days after transplantation. The median number of MSC infusions was 5 (4-6) times. Swimmer plot for the response was summarized in **Figure 1**, among the 16 patients, 13 patients (81.3%) had CR, 2 patients (12.5%) had PR, and 1 patient (6.3%) had NR. The median time to obtain CR was 32 (7-426) days after treatment with avatrombopag combined with umbilical cord MSCs. One patient started treatment with Eltrodopax 28 days after transplantation and did not reach CR at 373 days of treatment; treatment was subsequently changed to avatrombopag, and CR was achieved 53 days later. The time to reach 20×10^9/L≤ PLT<50×10^9/L in the 2 patients with PR was 52 and 230 days after treatment with avatrombopag combined with umbilical cord MSCs, respectively. One patient with NR had PLT<20×10^9/L, still relied on PLT transfusion, and died due to complications of infection and GVHD.

### Posttransplant Complications

#### Infection

Among the 16 patients, 10 patients had cytomegalovirus (CMV) infections, of whom 1 had CMV retinitis and retinal necrosis, 1 had an intracranial CMV infection, 1 had CMV infection combined with pulmonary infection, 2 had CMV infections combined with fungal infections, 1 had CMV infection combined with tuberculosis, and 1, who died, had CMV infection combined with pneumocystis carinii pneumonia and pulmonary infection. The other patients were treated with ganciclovir, sodium phosphonate injection, and intravenous gamma globulin to reduce the CMV copy number, and the infection was controlled after the administration of antibacterial, antifungal, and antituberculosis treatments.

#### GVHD

Among the 16 patients, 4 patients developed acute GVHD, including 2 cases of grade II GVHD and 2 cases of grade III GVHD, including skin rash, liver function damage, hematuria, nausea, and vomiting. Three patients were treated with 2 mg/kg methylprednisolone, and 1 patient had grade III GVHD combined with various complications, such as pulmonary infection and CMV infection, and was treated with sequential anti-GVHD treatments, including steroids, CD25 monoclonal antibody, and ruxolitinib. Eventually, this patient experienced multiple organ failure and intracranial hemorrhage and died at +108 days after transplantation.

#### Safety

Fourteen out of 16 patients had infections, including fungal infections, intracranial infections, and CMV infections. One patient had acute pancreatitis, 1 patient had pulmonary tuberculosis; and 1 patient had secondary hemolytic anemia. On the basis of clinical judgment, there was no correlation between the above symptoms and treatment with avatrombopag combined with MSCs. The drug was not discontinued due to adverse drug reactions or intolerance, and no thrombotic events occurred.

### Follow-Up

**Figure 2:**
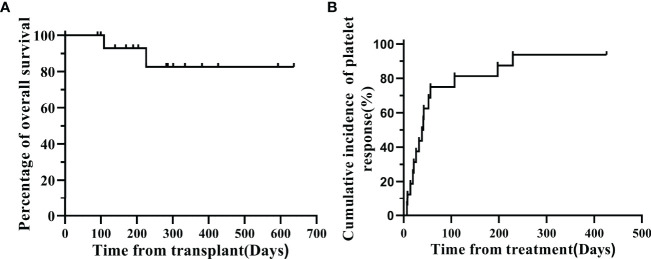
**(A)** Overall survival probability for the whole cohort. **(B)** cumulative incidence of platelet response.

During the follow-up until December 21, 2021, 11 of the 13 CR patients had stopped treatment with avatrombopag, and MSC infusions also had stopped. The median time from the start to the end of treatment was 81 (24-583) days, and the overall one-year survival was 82.54% (**Figure 2A**). The PLT count of all patients was maintained in the normal range. And the incidence of platelet recovery was summarized in **Figure 2B**, two patients with CR were treated for 26 and 57 days. For 2 patients, PR was maintained at (50~100)×10^9/L, and treatment was continued; 1 of the 2 PR patients discontinued treatment (total course of treatment with avatrombopag and MSCs was 52 days). Another patient had a total treatment duration of 242 days, with PLT count fluctuating between 20-30×10^9/L. The patient was not transfused with PLTs and was still undergoing treatment. One patient with NR died of pneumocystis carinii pneumonia, pulmonary infection, CMV, GVHD, and intracranial hemorrhage after 81 days of treatment.

## Discussion

Posttransplant thrombocytopenia is an independent risk factor for poor prognosis in patients with allo-HSCT. It has a high nonrelapse mortality rate and can increase the risk of infection events and adversely affect the long-term survival of patients (11). Zaja F et al. (12) found that in patients with a survival time of ≥3 months after HSCT, the mortality rate of patients with thrombocytopenia was significantly higher than that of patients without thrombocytopenia and that both primary and secondary thrombocytopenia after HSCT were correlated with a poor prognosis. The mechanism of thrombocytopenia after allo-HSCT is complex and involves multiple causes as follows: 1. Bone marrow microenvironment abnormalities – The production of megakaryocytes relies not only on a sufficient number of well-functioning HSCs but also on a stable bone marrow microenvironment. When the microenvironment of bone marrow cells is abnormal, the normal hematopoietic process is inhibited, resulting in a decrease in PLT production (13, 14). 2. Immune microenvironment abnormalities – The immune microenvironment of the bone marrow is an important factor affecting the production of megakaryocytes. Bone marrow is not only a hematopoietic organ but also an immune organ. Immune cells originate from HSCs and are a component of the hematopoietic microenvironment, which is regulated by the hematopoietic microenvironment and secretes cytokines to influence the hematopoietic process. Acute GVHD can inhibit hematopoiesis through cytokine storms and impair hematopoietic function, especially the development of megakaryocytes (15). 3. Abnormal PLT survival – Abnormal PLT survival includes reduced PLT production, increased PLT destruction and functional changes. Megakaryocytes interact with bone marrow endothelial cells to regulate the maturation of megakaryocytes and PLT production, and chemokines play an important role in the migration and differentiation of megakaryocytes. The expression of bone marrow endothelial cells and related chemokines in patients with thrombocytopenia after transplantation is impaired, leading to the occurrence of thrombocytopenia (13, 16).

Avatrombopag is a small molecule TPO receptor agonist that mimics the TPO effect and stimulates the proliferation and differentiation of megakaryocytes derived from bone marrow progenitor cells, thereby increasing PLT production. Avatrombopag interacts with the transmembrane domain of the human TPO receptor to initiate a signaling cascade, induce the proliferation and differentiation of bone marrow progenitor cells, and stimulate the production of PLTs by megakaryocytes. The ADAPT-1 and ADAPT-2 (17) studies found that for adult patients with chronic liver disease-associated thrombocytopenia who took avatrombopag (40 mg·d^-1^ or 60 mg·d^-1^ for 5 consecutive days, significant increase in PLT counts were observed at 3 to 5 days after the initial treatment; the PLT count peaked from 10 to 13 d, then gradually decreased and returned to the baseline level at 35 d. Michelson et al. (18) evaluated the effect of avatrombopag on platelet function, and the results indicated that avatrombopag did not reduce the PLT activation threshold while increasing the PLT count. In addition, Abe et al. (19) using cell proliferation experiments, found that the pharmacological activity of avatrombopag was more effective than that of eltrombopag and exerted greater *in vitro* pharmacological effects. Additionally, they also found that the oral administration of eltrombopag required a higher concentration to induce an increase in the PLT count and that the *in vivo* pharmacological efficacy of avatrombopag was greater than that of eltrombopag. Several studies have reported that the complete remission rate of patients with thrombocytopenia after transplantation rate was 50%-70% after treatment with eltrombopag (20–22). However, efficacy was still poor for some patients. Considering that the incidence of oral mucositis in patients undergoing transplantation is high, avatrombopag is not limited by food, absorption does not significantly decrease when taken with food, and variability in the pharmacokinetic indicators is lower when it was taken with food than in the fasting state (23, 24). In addition, the safety data for avatrombopag did not suggest a risk of hepatotoxicity (17), thus avoiding increased hepatotoxicity with immunosuppressive agents in patients who receive transplants.

As an important component of the bone marrow hematopoietic microenvironment, MSCs are important pluripotent stem cells that can secrete a variety of cytokines with that support hematopoiesis, can express a variety of adhesion molecules that interact with hematopoietic cells, and play important regulatory roles in hematopoietic reconstruction (25). MSCs can promote the proliferation and differentiation of HSCs by secreting a variety of cytokines, including granulocyte-macrophage colony-stimulating factor, granulocyte colony-stimulating factor, stromal cell-derived factor, and vascular endothelial growth factor (VEGF), thus enhancing hematopoietic function and supporting hematopoiesis (8). MSCs also directly participate in or indirectly promote the regeneration of damaged tissues and organs (26). They can secrete VEGF, which can promote vascular regeneration and improve blood circulation. A large number of clinical studies have found (27) that MSCs promote the homing, development, differentiation, and maturation of HSCs after transplantation as well as hematopoietic reconstitution after transplantation. Chen et al. (28) found that the interaction between MSCs and megakaryocytes contributes to the maturation of megakaryocytes and PLT production. Our previous study found that MSCs can prevent chronic GVHD in haploidentical HSCT (29). The synergistic effect of a TPO agonist (TPO-RA) and MSCs alleviates thrombocytopenia after transplantation.

We reviewed the cases of 16 patients with thrombocytopenia after allo-HSCT in our center, and 13 patients (81.3%) achieved CR. The CR rate was higher than that in previous reports on the efficacy of thrombocytopenia treatment after transplantation. Among the 3 patients with PT, 2 had aplastic anemia (AA). For this type of bone marrow failure, thrombocytopenia is associated with hematopoietic stem cell failure and bone marrow microenvironment disorders, but the hematopoietic microenvironment can be reconstructed through TPO-RA promoting PLT production and MSCs regulating immune function. The median time of thrombocytopenia in the 13 patients with SFPR was 63 (35-133) days after transplantation. Among the 16 patients, 10 patients had CMV infections, of whom 6 patients had CMV infections combined with other infections, such as bacterial and fungal infections. A retrospective study (30) of 342 patients with hematological malignancies who underwent HSCT showed that on the 50th day after HSCT, the PLT count of patients without CMV antigenemia was significantly higher than that of patients with CMV antigenemia. Akahoshi et al. (5) included 184 patients who underwent allo-HSCT for the first time and showed that the use of ganciclovir or valganciclovir increased the risk of SFPR in patients. The incidence of CMV infection in this group of patients was 62.5% (10/16). CMV infection is a common complication after allo-HSCT. CMV can directly inhibit hematopoiesis through bone marrow infection or indirectly inhibit hematopoiesis through stromal cell infection. CMV infects megakaryocytes and induces apoptosis, leading to a reduction in PLT production. Both CMV infection and its therapeutic drugs increase the risk of thrombocytopenia during HSCT. Therefore, it is very important to prevent and control CMV infection during the transplantation process to avoid the occurrence of thrombocytopenia caused by CMV infection.

Acute GVHD is a significant independent risk factor for the occurrence of secondary thrombocytopenia after transplantation, and grade III or IV GVHD significantly affects the recovery of the PLT count above 100×10^9/L 100 days after the transplantation (31). However, in this group of patients, only 4 patients had acute GVHD above grade II, including 2 cases of grade II GVHD and 2 cases of grade III GVHD, which resolved after treatment. Therefore, it is still not possible to determine the correlation between poor platelet recovery and GVHD in these 16 patients.

In this study, the median time to treatment efficacy was 32 (7-426) days for the 13 patients with CR, and the median number of MSC infusions was 5 (4-6) times. Eleven patients had stopped treatment, and all patients maintained a PLT count in the normal range. During treatment, 4 patients had acute pancreatitis, pulmonary tuberculosis, and secondary hemolytic anemia, events that were not significantly correlated with this treatment.

The high mortality rate for patients with thrombocytopenia after allo-HSCT should arouse high attention in clinical practice. The combination of avatrombopag and MSCs for the treatment of thrombocytopenia after transplantation has significant efficacy, good safety, and no obvious side effects, providing a novel first-line treatment. Future prospective and controlled studies are needed to further investigate the efficacy of this treatment on poor PLT engraftment after allo-HSCT.

## Data Availability Statement

The original contributions presented in the study are included in the article/supplementary material. Further inquiries can be directed to the corresponding author.

## Ethics Statement

The studies involving human participants were reviewed and approved by Xinqiao hospital Ethics committees. The patients/participants provided their written informed consent to participate in this study.

## Author Contributions

LeG conceived and designed the study. LDZ, PK, and LeG developed the methodology and analyzed and interpreted the data. LDZ and JL wrote the manuscript. JL, SCG, LW, LiG, HL, CZ, LeG, YF, TC, and XZ reviewed and revised the manuscript. All authors contributed to the article and approved the submitted version.

## Funding

This research was supported by the Chinese National Natural Science Foundation (No. 82170161), the Chongqing National Natural Science Key Foundation (cstc2019jcyj-zdxmX0023, cstc2020jcyj-msxmX0756) and the Research Fund from the Clinical Foundation of Army Medical University (2018JSLC0034, 2018XLC2012).

## Conflict of Interest

The authors declare that the research was conducted in the absence of any commercial or financial relationships that could be construed as a potential conflict of interest.

## Publisher’s Note

All claims expressed in this article are solely those of the authors and do not necessarily represent those of their affiliated organizations, or those of the publisher, the editors and the reviewers. Any product that may be evaluated in this article, or claim that may be made by its manufacturer, is not guaranteed or endorsed by the publisher.
